# Distal Ulna Excision for Giant Cell Tumor—Retrospective Functional Outcome and Recurrence Analysis from Single Tertiary Center

**DOI:** 10.1007/s13193-025-02355-z

**Published:** 2025-06-02

**Authors:** Saravana Kumar Jagannathan, Rajesh Bahadur Singh, Ayush Choudary

**Affiliations:** Department of Bone and Soft Tissue Oncology, HBCH & MPMMCC, Varanasi, India

**Keywords:** Giant cell tumor, Distal ulna, Wide excision, Reconstruction, Curettage, Wrist function

## Abstract

Giant cell tumor of bone (GCTB) is classified as an intermediate malignant bone tumor, and occurrences in the distal ulna are particularly rare. This study aims to evaluate the outcomes of treating GCTB in the distal ulna through wide excision. We conducted a retrospective analysis at a single center, focusing on patients with GCTB of the distal ulna. The study included six nonmetastatic patients (5 males, 1 female; mean age 31 years, range 19–48 years) who underwent wide excision of the tumor without reconstruction. All patients followed a physiotherapy protocol. The mean follow-up period was 20 months, ranging from 12 to 54 months. Mean resection length was 8.3 cm. There were no cases of local recurrence and distal metastasis. At the latest follow-up, mean grip strength was 81% compared to the normal side, mean Modified Mayo wrist score was 83, and mean Patient Rated Wrist/Hand Evaluation (PRWHE) score was 23. Ulnar carpal translation was not present. For giant cell tumors of the distal ulna, we recommend en bloc excision with adequate margins, irrespective of Campanacci grade, without the necessity for reconstruction.

## Introduction

Giant cell tumor of bone (GCTB) is defined as an intermediate malignant bone tumor by the World Health Organization in 2020 [1]. The tumor is known for its high recurrence rate and locally aggressive behavior, though it rarely metastasizes to the lungs. It predominantly affects young adults aged 20 to 40, with a slight predilection for females [1, 2].

Patients with GCTB typically present with pain, swelling, and loss of function, which vary based on the tumor’s size and location. Diagnosis involves plain radiography, CT, and MRI, with biopsy necessary for confirmation. The presence of mutated H3 F3 A serves as a specific marker for GCTB, aiding in the differentiation from other similar lytic bone lesions. The most common sites for GCTB occurrence are the femur, tibia, and radius [2]. The distal ulna is a rare location accounting for 0.45% to 3.2% of all cases of GCTB [3]. These patients present with swelling, pain over the wrist, reduced wrist function, pathological fractures, and occasionally an exophytic mass.

Surgery is the preferred form of treatment for GCTB. Campanacci classification guides treatment; traditionally, grade 1 and grade 2 lesions are treated with intralesional extended curettage, while grade 3 lesions require en bloc resection and reconstruction, sometimes supplemented with denosumab. The distal ulna has a thin cortex and high chances of cortical breach due to the lytic action of GCTB. A high incidence of local recurrence has been reported following intralesional curettage. Hence, in all Campanacci grade lesions in the ulna are recommended to undergo en bloc excision [4, 5]. Some studies suggest that loss of bone and soft tissue support in the distal forearm following en bloc excision might cause instability, chronic pain due to ulnar stump impingement, carpal translation, and DRUJ disruption, and advise reconstructive procedures [6–14]. However, some studies favor no additional reconstruction procedures. Hence, this study was undertaken to analyze whether reconstruction is necessary after en bloc excision of distal ulnar GCTB.

## Materials and Methods

With approval from the institutional ethics board, a retrospective analysis was conducted on 6 patients over 18 years of age (male female ratio 5:1; mean age 31 years, range 19–48 years) with biopsy-proven localized giant cell tumor of the distal ulna. Patients with multiple site lesions, exophytic lesions, or lung metastases were excluded from the study. The surgeries were performed between January 1, 2020, and August 31, 2023, with a minimum follow-up of 1 year.

Upon arrival, patients were evaluated by X-rays and MRI of the entire forearm with wrist and elbow joints. Then, the core needle biopsy was taken along the line of incision of the definitive procedure to facilitate the resection of the biopsy scar along with the tumor specimen. Chest X-ray was taken to rule out any pulmonary metastasis. Serum calcium and serum phosphorus levels were measured to rule out Brown’s tumor/Hyperparathyroidism. Campanacci grading was done by radiological assessment. The size of the lesion was evaluated via MRI. For patients with extensive soft tissue extension, adjuvant therapy with denosumab was initiated. Our dosage regimen is denosumab 120 mg subcutaneous on days 0 and 14. On day 28, patients were examined clinically for improved wrist function, swelling, and pain reduction. Once the lesion showed containment by peripheral sclerosis, surgery was performed.

One patient initially underwent curettage and iliac crest bone grafting by a general orthopedic surgeon but presented with recurrence. The remaining 5 patients received primary treatment at our institution. Of these, one patient with a pathological fracture was given slab support and denosumab. Four patients received denosumab as adjuvant therapy. Two patients underwent upfront wide excision upon confirmation of GCTB.

A longitudinal incision was made along the distal ulnar subcutaneous border, including the biopsy scar. The ulnar artery, ulnar nerve, and uninvolved muscle tendons were carefully resected away from the tumor. An appropriate length of the ulna was resected with a 1–2 cm margin of bone and soft tissue, as planned with the pre-denosumab MRI. The pronator quadratus and the capsule of the distal radio ulnar joint were kept as soft tissue cover over the tumor and dissected along with the tumor. No additional procedure was performed to reconstruct the bone defect following resection. The remaining capsule was sutured to the adjacent soft tissue. Postoperatively, an ulnar gutter splint was applied for pain relief and to promote early soft tissue healing for 6 weeks. Finger and elbow movements were encouraged as tolerated. After 6 weeks, a removable splint was advised for an additional 6 weeks, along with intermittent wrist and forearm exercises.

Pathological analysis of the specimen was performed to evaluate tumor involvement at the bone and soft tissue cut margins. Patients were followed every 6 weeks for the first 6 months, and subsequently every 3 months. Follow-up included X-rays of the forearm and wrist, as well as a chest X-ray to monitor for local recurrence and distant metastasis. Functional assessment was conducted using the Modified Mayo Wrist Score (MMWS), Musculoskeletal Tumour Society Score (MSTS93), Patient Rated Wrist/Hand Evaluation (PRWHE), and grip strength [15–18]. Range of movements was measured with a goniometer. Hand grip strength was assessed using a handheld dynamometer and expressed as a percentage of the grip strength on the opposite normal side and adjusted for hand dominance (10% deduction for right-hand dominance and no deduction for left-hand dominance, based on published normative values) [16]. By Gilula method, we measured ulnar carpal translocation. Ulnar translocation is considered present when 40% or more of the lunate is uncovered by the radius on a posteroanterior view of the wrist [19].

## Results

**Fig. 1:**
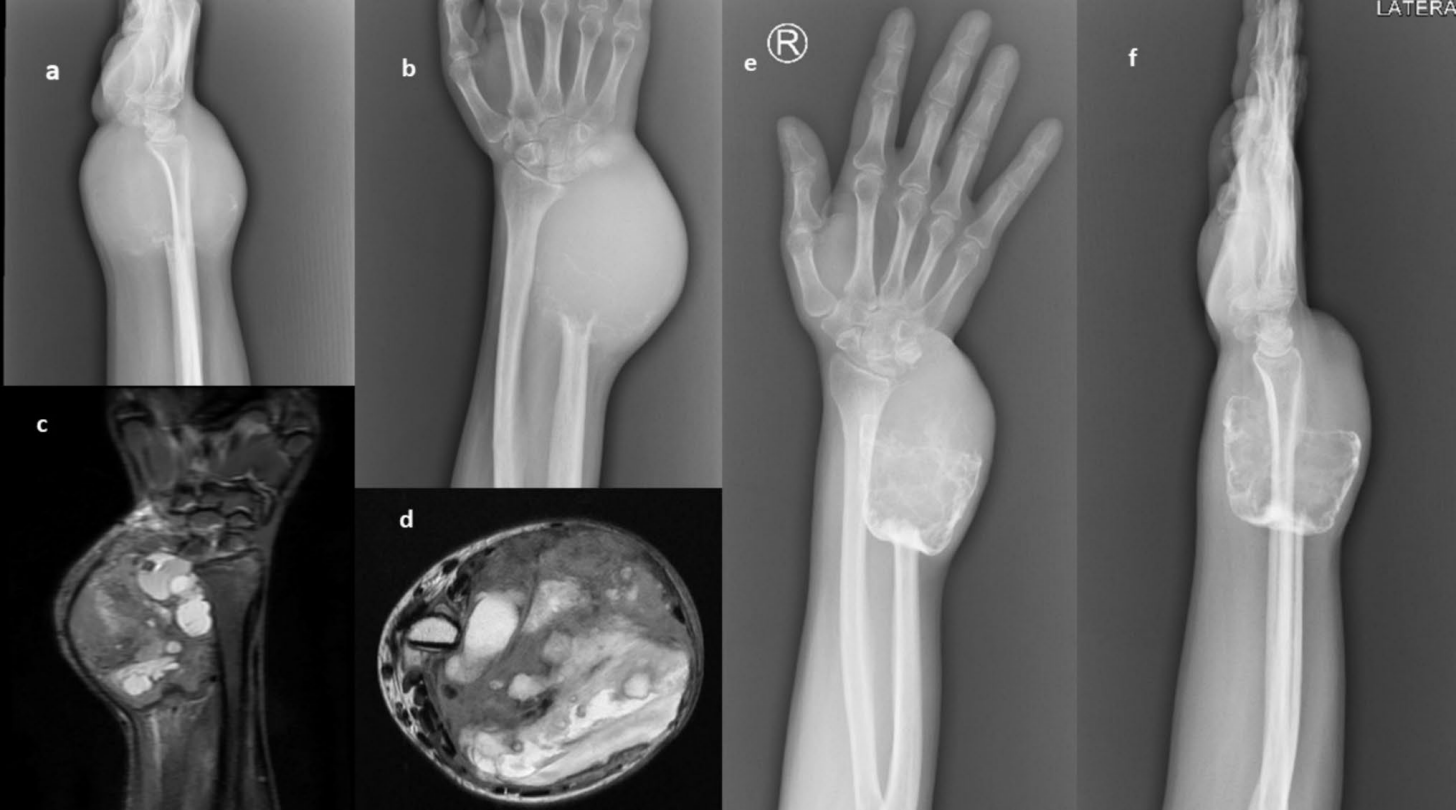
On initial presentation, **a**, **b** X-ray images showing eccentric lytic lesions and **c**, **d** MRI images showing soft tissue extension with cystic component. **e**, **f** X-ray images after denosumab adjuvant therapy with intralesional and peripheral sclerosis

**Fig. 2:**
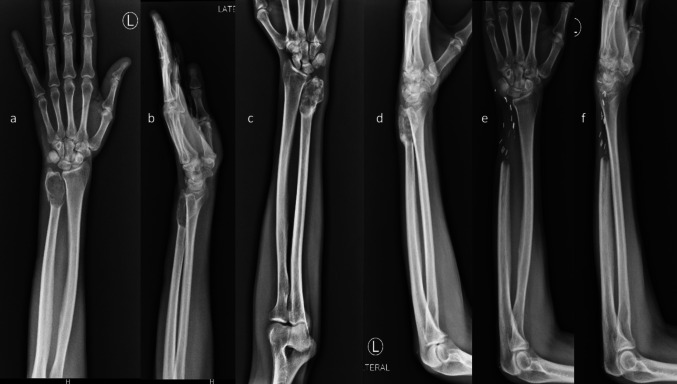
X-ray images **a**, **b** showing pathological fracture of ulna on initial presentation, **c**, **d** fracture union on denosumab adjuvant and pop slab support, and **e**, **f** 1-year follow-up X-ray

Six patients were operated on during the study period (Table 1). The median age was 31 years, with a range of 19 to 48 years. Of these, 5 patients had primary tumors and 1 had a recurrent tumor. The mean longitudinal length of the lesions was 7.6 cm, with a range of 5 to 13 cm. Campanacci grade was 2 in one patient who underwent upfront excision, while the remaining 5 patients had grade 3 tumors. Four of these 5 patients received neoadjuvant denosumab (Fig. 1). One patient with a pathological fracture at presentation healed with 3 doses of denosumab (Fig. 2). Another patient, a student, received 4 doses of denosumab to delay surgery due to ongoing examinations. Two patients received 2 doses of denosumab. Following denosumab therapy, surgery was performed 6 to 20 weeks later, with a mean interval of 13 weeks. Bone and soft tissue cut margins were free from disease in all the specimens. Mean resection length was 8.3 cm. The mean follow-up duration was 20 months, with a range of 12 to 54 months.

**Table 1:** Demographic and characteristic data of distal ulna GCT patients

S. No	Age/sex	Side	Pri/Rec	Campanacci grade and largest dimension on presentation	Denosumab doses	Excision length Cm	Follow-up Months
1	32/F	Dominant	Pri	Grade 3, 7 cm	-	9	54
2	47/M	Dominant	Rec	Grade 3, 13 Cm	2	13	16
3	21/M	Non-dominant	Pri	Grade 3, 9 Cm	4	9	15
4	19/M	Dominant	Pri	Grade 2, 7 cm	-	10	15
5	19/M	Non-dominant	Pri	Grade 3, 5 Cm	2	6	13
6	48/M	Non-dominant	Pri	Grade 3, 5 Cm	3	6	12

*Pri* primary, *Rec* recurrent, *ICBG* iliac crest bone grafting

**Fig. 3:**
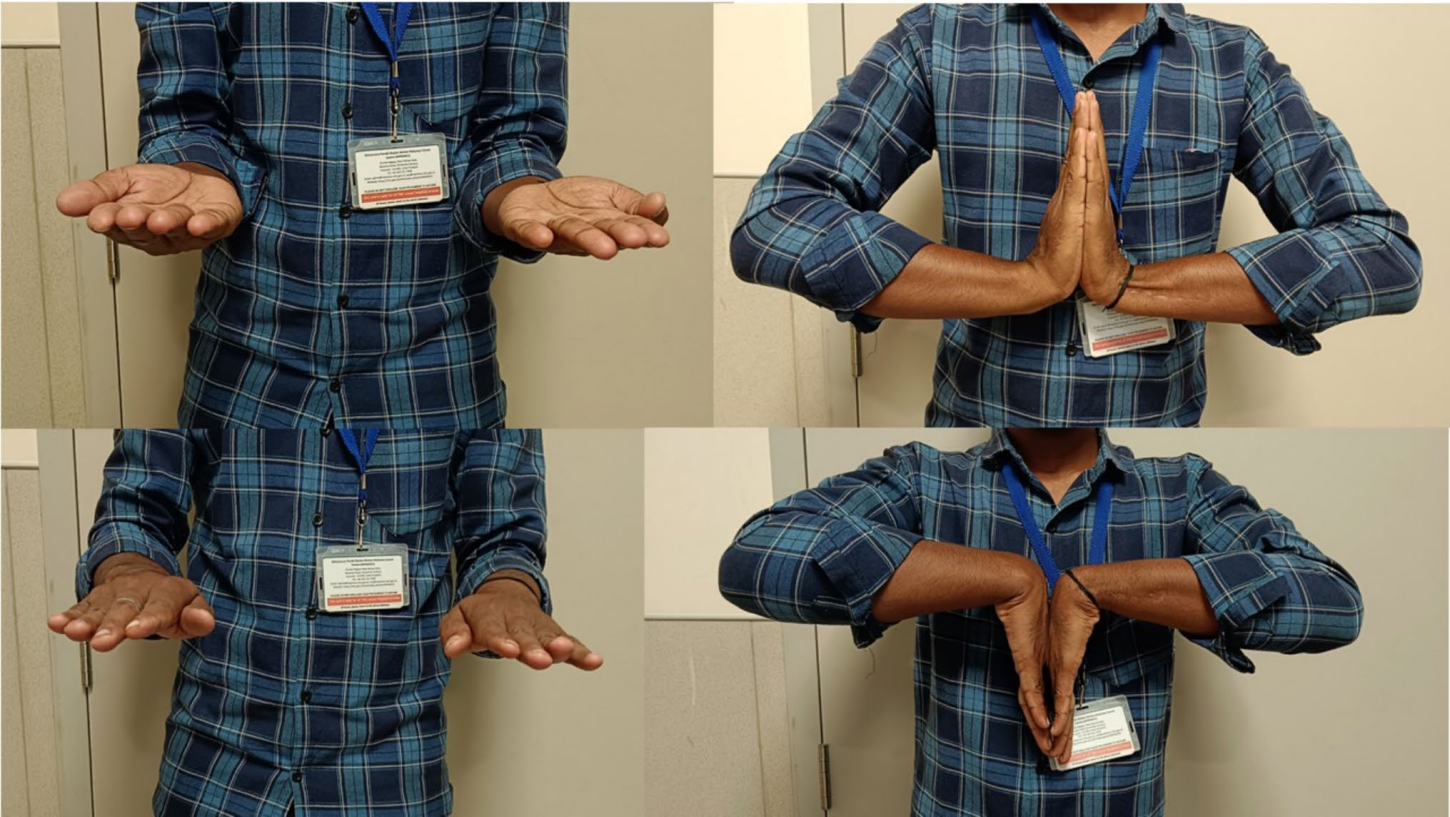
Clinical images showing wrist functions. Left side operated with visible surgical scar on 15-month follow-up. Supination, wrist dorsiflexion, pronation, and palmar flexion of operated left side are equal with the right side

None of the patients experienced non-oncological complications such as infection, chronic pain, or noticeable deformity. Two patients reported occasional pain when lifting heavy objects. Minimal skin puckering, a lean wrist, and a prominent extensor carpi ulnaris tendon were a concern for 2 patients but were easily managed by them with a full sleeve shirt or wrist band. No local recurrence and distant metastasis were observed. At the latest follow-up (Table 2), the mean grip strength was 81% compared to the normal side (range 78–85), mean MMWS was 83 (range 75–90), mean MSTS93 score was 27 (range 25–30), and mean Patient Rated Wrist/Hand Evaluation (PRWHE) score was 23 (range 14–33). Range of motion was minimally restricted compared to the opposite side in all planes (supination, pronation, flexion and extension) (Fig. 3). Minimal restriction of radio ulnar arc was observed. Ulnar carpal translation was not present in any patient. Lunate uncovering was less than 40% in all the patients. No patients exhibited wrist instability, ulnar subluxation, ulnocarpal translocation, or ulnar angulation of the wrist.

**Table 2:** Functional outcome

S. No	Grip strength (% of opposite side)	Restriction of wrist flexion/extension (degrees)	Restriction of supination/pronation (degrees)	Restriction of ulnar-radial deviation arc (degrees)	MMWS (%)	MSTS 93 score	PRWHE score
1	80	0/5	5/5	5	80	26	21
2	78	5/0	10/10	10	75	25	33
3	80	5/10	5/0	0	85	26	24
4	84	0/0	5/0	5	85	28	25
5	79	0/0	0/5	0	90	30	14
6	85	5/5	0/0	5	85	27	21

*MMWS* Modified Mayo Wrist Score, *MSTS93* Musculoskeletal Tumour Society Score, *PRWHE* Patient Rated Wrist/Hand Evaluation

## Discussion

Giant cell tumor is a primary bone tumor that typically occurs in long bones, accounting for 85% of cases, with 10% found in the axial skeleton [1]. It commonly presents at the epiphysis of long bones. Histologically, GCTB is characterized by neoplastic mononuclear stromal cells interspersed with macrophages and osteoclast-like giant cells. The incidence of GCTB is notably higher in Asian populations, with the highest reported rate in South India at 30.3% [20]. GCTB of the distal ulna is rare, with an incidence of 0.45–3.2% [3].

In long bones, GCTB Campanacci grade 1 and 2 lesions are managed with extended curettage with various adjuvants like hydrogen peroxide, argon laser, phenol, or liquid nitrogen and followed by reconstruction with bone graft or polymethylmethacrylate. For grade 3 lesions, excision is generally recommended, with or without the use of denosumab. The pathogenesis of GCTB involves the RANK/RANKL interaction, which is crucial for tumor development. Denosumab, a monoclonal antibody targ9eting RANK ligand, has a downstaging effect on GCTB, aiding in treatment and management. Similar to distal ulna, proximal fibula, metacarpals, and metatarsals have a thin bony cortex. This often leads to early bone erosion and significant soft tissue extension by GCTB. Even in Campanacci grades 1 and 2, performing extended curettage is challenging due to the thin cortex, leading to a high risk of recurrence [4, 21].

Distal ulna, ulnar styloid process, and triangular fibrocartilage complex (TFCC) are essential for providing stability to the wrist during forearm movements, hand gripping, and weight-bearing activities like weightlifting. In traumatic cases, triangular fibrocartilage complex (TFCC) lesions cause chronic pain due to mechanical derangement. Hence, anatomical reduction of ulnar styloid and TFCC repair is recommended [22]. Even in arthritic disorders of the wrist, excision of distal ulna causes various complications. Hence, various reconstruction procedures were advised to overcome musculoskeletal derangement [6–14]. However, in tumors of distal ulna, complete excision does not significantly disrupt wrist mechanics, as major wrist functions are performed at the radiocarpal joint [23–25]. And forearm rotation is also not compromised as there is no obvious mechanical hindrance.

Darrach popularized distal ulna excision for degenerative conditions [26]. Many authors post excision observed complications like carpal translation, impingement, instability, etc. [6]. Hence, several soft tissue and bony reconstruction surgical procedures have been described following en bloc resection of distal ulna [6–14]. Goldner and Hayes first described dynamic stabilization of the ulnar stump by ECU tendon strip post excision of the distal ulna [11]. Breen et al. in arthritic distal radio ulnar joint after the Darrach procedure, achieved stability by tenodesis of the ECU and FCU tendons [12]. Johnson described a surgical procedure for distal ulna stabilization by transfer of the pronator quadratus origin [13].

Various bony reconstruction procedures are also described in the literature after the excision of distal ulna tumors. Hashizume et al. stabilized the wrist by ulnar buttress arthroplasty. In this procedure, they used iliac crest bone graft for fixation [14]. Kapoor et al. in their recent study utilized this method and achieved excellent hand functions [10]. Complete distal radioulnar allograft reconstruction was achieved by Wurapa and Whipple [27]. Stoffelen et al. utilized segmental bone transport using the Ilizarov fixator to fill the bony defect [28]. Even high-cost DRUJ arthroplasty was attempted and achieved excellent results in the short term [29, 30].

There were no local recurrences and distant metastases observed in our study. The primary goal during oncological surgery is to prevent tumor spillage and ensure clear bone and soft tissue margins. Repeat surgeries are associated with increased morbidity due to excessive resection and a higher risk of both local and distant tumor recurrence. Cooney et al. reported that eight patients who underwent en bloc resection of the distal ulna experienced no recurrences over a 2-year follow-up period [5]. Jamshidi et al. found that in cases of grade 3 giant cell tumor of the distal ulna, curettage resulted in a 100% recurrence rate, whereas en bloc excision had no recurrence [21]. Therefore, en bloc excision is recommended for all Campanacci grade lesions of the distal ulna [5, 21, 31].

Errani et al. found limb function following en bloc resection of the proximal fibula and distal ulna is generally good, and they recommended en bloc resection for GCTB in these locations [23]. Papanastassiou et al. in their study compared extensor carpi ulnaris tenodesis against no stabilization after resection of distal ulna GCTB. Both the groups achieved similar functional outcomes, and they suggested a reconstruction procedure is not mandatory following GCTB ulna excision [24]. Wolfe et al. in their multi-center case study following distal ulna excision without any additional procedure, patients achieved 75% of grip strength and 86% of range of movements compared to the normal side [25] similar to our study results.

Many authors have documented that en bloc resection of the distal ulna, in degenerative conditions, can lead to dorsal translation of the ulna at the resection site during pronation and often results in a painful stump or instability [6–9]. But all of our patients were able to achieve painless wrist and forearm movements by adhering to our physiotherapy protocol. Similarly, Cooney et al. reported excellent results in six out of eight patients who underwent en bloc resection of the distal ulna without any mode of reconstructive procedures [5]. Jamshidi et al. in their study with 5 distal ulna resections observed good wrist function without any reconstruction [21]. Love Kapoor et al. in their study analyzed ulnar buttress arthroplasty following excision of distal ulna GCTB in 8 patients with a mean follow-up of 3 years. They found mean grip strength was 90% comparable to the nonoperative side. Mean MSTS93 score was 27.9, mean MMWS was 86.9%, and the mean DASH score was 4.9 with good functional outcomes. Comparing their study, our patients have a better range of movements at the wrist with comparable grip strength of 81% [10].

There are several limitations in this study. They are short-term follow-up period, retrospective study design, and lack of comparative group. Additionally, the study population is small due to the rarity of giant cell tumors at the distal end of the ulna. It is also important to note that oncological patients typically have fewer functional demands compared to trauma patients, which may influence the outcomes observed.

## Conclusion

For grade 3 Campanacci giant cell tumors of the distal ulna, we recommend en bloc excision with adequate margins, without the need for reconstruction. Post-surgery, patients should undergo comprehensive physiotherapy to promote soft tissue healing and muscle strengthening. Further research is needed to evaluate the effectiveness of wide excision in both benign and malignant tumors affecting the distal ulna and to explore the application of these principles to traumatic distal ulnar conditions when reconstruction is not feasible.

## Data Availability

Data is available with the corresponding author and will be provided at request.
